# Exploration of health care utilization, social care utilization and costs for individuals discharged from inpatient geriatric care in Sweden - a registry data study

**DOI:** 10.1186/s13561-025-00610-1

**Published:** 2025-03-13

**Authors:** Carl Willers, Rikard Lindqvist, Stefan Fors, Amelie Lindh Mazya, Gunnar H Nilsson, Anne-Marie Boström, Elisabeth Rydwik

**Affiliations:** 1 Division of Physiotherapy, Department of Neurobiology, Care Sciences and Society, Karolinska Institute, Huddinge, Sweden; 2 Stockholm Region Council, FOU nu, Research and Development Center for the Elderly, Järfälla, Sweden; 3 Division of Nursing, Department of Neurobiology, Care Sciences and Society, Karolinska Institutet, Stockholm, Sweden; 4 Aging Research Center, Department of Neurobiology, Care Sciences and Society, Karolinska Institutet & Stockholm University, Stockholm, Sweden; 5 Centre for Epidemiology and Community Medicine, Region Stockholm, Stockholm, Sweden; 6 Division of Clinical Geriatrics, Department of Neurobiology, Care Sciences and Society, Karolinska Institutet, Stockholm, Sweden; 7 Geriatric Department of Danderyd Hospital, Stockholm, Sweden; 8 Division of Family Medicine and Primary care, Department of Neurobiology, Care Sciences and Society, Karolinska Institutet, Huddinge, Sweden; 9 Academic Primary Care Center, Stockholm Region Council, Stockholm, Sweden; 10 Theme Inflammation and Aging, Karolinska University Hospital, Huddinge, Sweden; 11 R&D unit, Stockholms Sjukhem, Stockholm, Sweden; 12 Medical Unit for Occupational Therapy and Physiotherapy, Karolinska University Hospital, Theme Women’s Health and Allied Health Professional, Solna, Sweden; 13 Department of Public Health Sciences, Stockholm University, Stockholm, Sweden

## Abstract

**Background:**

Individuals receiving geriatric care are often frail and afflicted with multiple chronic conditions demanding care from several medical disciplines, and from several different care providing units across the health systems.

**Objective:**

To explore the six-month service utilization and direct costs attributed to individuals receiving geriatric inpatient care.

**Methods:**

Health care utilization– in terms of inpatient care, outpatient visits with different health care professions– and social care utilization– in terms of social services, and stay at residential care facility (RCF)– were quantified based on registry data, for a cohort admitted to geriatric inpatient care in Stockholm, Sweden during 2016.

**Results:**

On average, individuals admitted to geriatric inpatient care in Stockholm had 12.8 inpatient care days, 32.1 visits in outpatient care, 432 h of home care and 28.8 days of staying at RCF, during the first six months after discharge. This amounted to an average cost of 722 thousand Swedish kronor (SEK), € 64 900, in 2023 monetary value. Costs were on average 37% attributable to inpatient care including the initial inpatient stay, 12% to outpatient visits, 38% to social services at home, and 13% to stay at residential care facility (whereof 11% short-term, 89% long-term). Total costs differed significantly between groups based on function, age and main diagnosis.

**Conclusion:**

Costs of care for individuals treated at geriatric department are substantial. The variation of cost is also significant; higher age and lower function were associated with higher health care and social care costs. Major cost buckets were inpatient health care (region-financed) and social care at home (municipality-financed).

**Supplementary Information:**

The online version contains supplementary material available at 10.1186/s13561-025-00610-1.

## Introduction

Economic evaluation is an important concept in health care as it enhances the understanding of service utilization and builds a basis for resource allocation. Hence it is essential for making informed decisions regarding prioritization of available resources between different health conditions as well as between different ways of working for a given medical condition. Such decisions demand knowledge on the most relevant outcomes but also on their underlying costs, and to enable adequate cost analysis it is necessary to use reliable data on service utilization.

Individuals in need of geriatric inpatient care require, to a larger extent than other groups, post-discharge support (formal or informal) concerning personal activities of daily living (pADL) such as hygiene, support with eating, toileting, mobility, and medical care as well as instrumental activities of daily living (iADL) such as cleaning, food preparation and laundry. This includes home-based health care, home-care services and support, or moving to a residential care facility (RCF), either a short-term or a long-term care facilityif support at the ordinary home is not sufficient considering their needs. Needs fulfilled by home-care services include personal care (e.g. shower) and other services (e.g. grocery shopping) [1]. In the context of this study it is of relevance to separate medical and social services which also have different financers; the region finances health care, at care facility or at home, and the municipality finances home-care services and stay at RCF, together referred to in this paper as social services.

Available literature on service utilization or costs of care is generally focused on particular conditions or interventions, and individuals in need of geriatric inpatient hospital care in general, is not extensively studied compared to other groups [2]. Furthermore, studies on utilization and costs of home-care services seem even more scarce [3]. It is unfortunate since this patient group is often afflicted with multiple medical conditions, polypharmacy [4], and frailty [5], therefore overall likely implying significant cost. Inappropriate medication use has been covered extensively [6], as well as burden of disease for particular health conditions and costs relating to specific interventions and pharmaceuticals [7–9]. Service utilization has also been subject to recent studies on older adults with psychiatric comorbidity [10, 11]. However, availability of studies on individuals in need of geriatric care or older adults in general and their care services utilization is not as common although they do exist [12, 13].

Relatively fewer examples of service utilization and cost studies for this patient group compared to others suggests an inequity; low availability of granular cost analysis means that the possibilities to perform evaluations of new interventions or updated ways of working for this group are limited. Thus, there is a risk that advancement of care for this group, fails to appear, or– best-case– fails to be properly analyzed. A better understanding of the cost incurred for care of individuals treated in geriatric inpatient care would improve the grounds on which to perform further health economic analysis, such as evaluation of cost effectiveness for new care pathways and updated ways of working. This should include the understanding of costs attributable to care throughout the full continuum of care, including inpatient and outpatient care as well as services at home or in the RCF context. The present study was initiated to enable a full such assessment of utilization and cost of formal care for individuals with a recent history of geriatric inpatient care.

## Aims

The aim was to explore utilization attributable to publicly financed health care and social care, and their costs, for individuals admitted to geriatric inpatient care. Furthermore, the aim was to assess costs for different groups based on age, physical function and main diagnosis at enrollment in inpatient care.

## Materials and methods

### Setting and sample

In Sweden in general, health care in hospital and primary care is financed by the region (21 regions in total), and social care and health care in RCF as well as home health care is financed by the municipality (290 municipalities in total). The services– health care or social care– may be executed by a publicly financed provider or by a private provider which is either a ‘for-profit’ or ‘not-for-profit’ organization. The patient group studied here, i.e. qualifying for geriatric hospital care, generally has a need for services financed by the region as well as by the municipality.

Health care includes inpatient care (at hospital) and outpatient care, offered at a hospital, a primary care centre or at home. Social care may include either delivering home-care services such as help with personal hygiene, grocery shopping or cleaning, or offering a stay at an RCF as a temporary or long-term alternative to the patient’s ordinary home. The region finances health care whilst the municipality finances social care. Health care delivered at social care facilities (RCF) is financed by the municipality, except for doctor visits, whilst home-based health care (at the individual’s ordinary home) is financed by the region in Stockholm (as stated above, this is not the case for the rest of Sweden) [14]. The study population of the present study was admitted to geriatric inpatient care in Stockholm, the largest region in terms of inhabitants (2.2 Million in 2016 [15]), and encompassing 26 different municipalities. There are approximately 40 specialized geriatric care departments throughout the country, whereof 15 are situated in the Stockholm region. The target individual of geriatric care has been defined as biologically aged, with functional impairment, and who is dependent on others in their daily living or suffering simultaneously from more than one serious health condition [14, 16].

The study population included all individuals admitted to geriatric inpatient care at either of three publicly operated geriatric departments in the Stockholm region during 2016. The individual’s last inpatient admission to either of these three clinics during that year is hereafter referred to as the index admission. Data on region-financed health care and municipality-financed social services were available during the six months after discharge from the index admission. Individuals who formally resided in other regions during the index admission or who moved from the Stockholm region during follow-up were excluded from the analysis (supplemental Figure S1). The study population and its context have been described in previous publications [17, 18].

### Data sources

The present study includes a broad spectrum of data for a cohort of individuals admitted to geriatric inpatient care. Four different sources of individual-level data were leveraged, together with cost data that were available on aggregated level; (1) the electronic health records (EHR) including data on the admission at geriatric clinic, i.e. all diagnoses registered and risk screening scores at admission and at discharge; (2) the health care administrative register (VAL) including information on additional inpatient admissions as well as all outpatient care including home-based health care; (3) data extracts from the population databases at Statistics Sweden which contains data on socioeconomics, co-habiting status, marital status and region of birth to mention a few; (4) data from the registry for care and social services support for older adults and for persons with impairments (SoL) which is run by the National Board of Health and Welfare, containing information on utilization of services offered by the municipality, such as home-care services and stay at RCF, and (5) the Cost per patient (CPP) and Cost per user (CPU) databases were leveraged for information on actual costs. These costing databases are run by the Swedish National Board of Health and Welfare and available online [19]. The cost data are based on reporting from the regions and the municipalities in Sweden. Coverage rates are high for CPP (e.g. approximated to 94% for inpatient care) but not as high regarding CPU [20]. The data refer to actual (direct and indirect) costs, and are based on time-driven activity-based costing estimations performed by the reporting units [21].

The individuals’ personal identity numbers were pseudonymized, meaning that the researchers were not able to identify individual patients. The pseudonymization number did however enable linking data between the data extracts to perform individual-level analysis.

Ethical approval was granted for this research by the Regional Ethical Review board in Stockholm (reference numbers 2013 − 1620/31/2; 2018/247 − 32) and by the Swedish Ethical Review Authority (reference number 2019–02837). Data were analyzed using SAS 9.4 and Microsoft Excel.

### Variable definitions and assumptions

Patients were categorized by physical function to enable cost analysis per category of ability relating to ADL with the modified Barthel index. The index was initially developed for stroke patients but has since been validated and used for broader, more heterogenous patient groups, also with regards to health economic analysis [22–25]. Cut-off values to categorize according to the Barthel index were *≤* 20 (total dependency), 21–60 (severe dependency), 61–90 (moderate dependency), 91–100 (slight or no dependency), in accordance with previous research [22].

Patients were also categorized to enable cost analysis per health condition; eight of the most frequently registered diagnoses in the study population were presented separately. Categorization was based on main diagnosis during the index admission and performed using the Swedish version of the International Classification of Diseases, 10th Revision (ICD-10-SE), codes [26]. The sets of codes applied are presented in Table S1 in the supplement. Patient categorization was also performed based on age groups.

Baseline characteristics presented for the study population include demographics (sex, age), morbidity (number of diagnoses registered, number of pharmaceuticals prescribed for continuous intake), physical function (Barthel activities of daily living (ADL) index, the Rivermead mobility index (RMI)), risk screening instrument scores (the Downton fall risk index, the Norton pressure ulcer risk screening score, the mini nutritional assessment (MNA) score), and socioeconomics (educational level, disposable income, region of birth, marital status and co-habiting situation). The risk screening instruments are used in clinical practice and their scores were presented as binary variables; Downton fall risk index (0–11) where *≥* 3 was considered high fall risk [27], the modified Norton pressure ulcer risk screening score [5–28] where *≤* 20 was considered high risk [28], and the mini nutritional assessment (MNA) score (0–14) where *≤* 11 was considered as risk for malnutrition [29].

### Service utilization

Calculations on service utilization were based on care days during index admission, care days during additional admissions at any type of hospital clinic, outpatient visits including specialty and primary care, and social home-care services and stay at RCF. Information on service utilization was available on a daily level for EHR and VAL (health care), and on a monthly level for SoL (social care). Data on outpatient visits included information on health care personnel category as well as type of facility the visit took place at. Individuals who had been granted stay at long-term care facility were assumed to stay at such during the months there were registrations, and they were assumed to do so for each full month. Individuals who had been granted a short-term care facility stay was assumed to stay there for the total amount of registered number of days.

### Estimation of costs

Costs refer to the total costs related to the event from the care financers’ point of view, with all fixed and variable costs incurred by the care giving unit. Only costs incurred by the care giving units were included. To calculate actual costs attributable to each activity, service units– inpatient care days, outpatient visits, home-care service hours, and days of staying at RCF– were multiplied by unit costs available from the Cost per patient (CPP) and Cost per user (CPU) databases (Table 1).

For inpatient and specialized outpatient care, figures reported for Stockholm were leveraged, and for primary care, figures for Stockholm were not available and hence figures from the regions Uppsala and Södermanland in close geographical proximity were used instead. This may alter estimations of costs for home-based health care for individuals living in RCF, as primary care facilities in Stockholm are responsible for it whilst this is not the case for any other regions, including Uppsala and Södermanland.

Costs were enumerated with consideration to inflation, to 2023 values, and the SEK/EUR exchange rate per December 31 2023 (11.13) was used [30, 31]. Indirect costs outside of the formal health care system were excluded, as available estimates are uncertain.

**Table 1:** Unit costs for computation of monetary cost, from Cost-Per-Patient and Cost-Per-User databases available from the National board of health and welfare. Costs reported for Stockholm were used except for primary care where cost data for Södermanland and Uppsala were combined as Stockholm data were missing. Outpatient specialty care includes hospital-based and non-hospital based specialty care. Costs for team visits based on assumption that the team most commonly consisted of a nurse, a physiotherapist (PT), and an occupational therapist (OT)

	Unit cost (2023)	
	SEK	€
Inpatient stay (day)	19 453	1 748
Outpatient - specialty care - doctor (visit)	5 805	522
Outpatient - specialty care - doctor (home visit)	5 488	493
Outpatient - primary care - doctor (visit)	2 442	220
Outpatient - primary care - doctor (home visit)	3 173	285
Outpatient - specialty care - nurse (visit)	4 034	363
Outpatient - specialty care - nurse (home visit)	6 340	570
Outpatient - primary care - nurse (visit)	1 108	100
Outpatient - primary care - nurse (home visit)	1 702	153
Outpatient - specialty care - OT (visit)	2 656	239
Outpatient - specialty care - OT (home visit)	2 756	248
Outpatient - primary care - OT (visit)	1 252	113
Outpatient - primary care - OT (home visit)	2 787	251
Outpatient - specialty care - PT (visit)	3 252	292
Outpatient - specialty care - PT (home visit)	1 555	140
Outpatient - primary care - PT (visit)	1 483	133
Outpatient - primary care - PT (home visit)	2 085	187
Outpatient - specialty care - assistant nurse (visit)	2 865	258
Outpatient - specialty care - assistant nurse (home visit)	495	44
Outpatient - primary care - assistant nurse (visit)	1 303	117
Outpatient - primary care - assistant nurse (home visit)	1 089	98
Outpatient - specialty care - other health personnel (visit)	3 978	358
Outpatient - specialty care - other health personnel (home visit)	4 739	426
Outpatient - primary care - other health personnel (visit)	2 069	186
Outpatient - primary care - other health personnel (home visit)	3 226	290
Team visit - specialty care	10 296	925
Team visit - primary care	5 208	468
Social service (hour)	586	53
Residential care facility (day)	2 938	264

### Statistics

Baseline characteristics are presented as mean and standard deviation (continuous variables), or as number of observations and percentage (categorical variables). Service utilization is presented per service category, and as mean with a 95% confidence interval.

To assess differences between groups in terms of total costs, ANOVA models were used for multiple comparisons between groups based on age, based on Barthel score, and based on main diagnosis. These analyses were made post-hoc. Results were considered statistically significant at the 5% level (*p* < 0.05).

## Results

### Baseline characteristics

Characteristics of the study population are presented in Table 2 (*n* = 8,067; in total 37 individuals were excluded due to not residing in the region and/or not available in all registers). Almost 63% were women, and average age at baseline was 83.5 years. The ADL score according to the Barthel index amounted on average to 52 out of 100 points (97 points in the subgroup with lowest ADL dependency, and 11 points in the group with highest ADL dependency). All risk screening measures (RMI, proportions with Downton *≥* 3, Norton *≤* 20 and MNA *≤* 11 respectively) pointed to higher risks with increasing age as well as with increasing degree of ADL dependency.

The socioeconomic indicators showed similar levels across groups of physical function. For the different age groups, there were differences including proportions having finished only primary school, which were smaller in the youngest age group (< 70 years), and the proportions having finished post-secondary and higher post-secondary which were smaller in the oldest age group (> 90 years). Furthermore, the proportion of never married decreased with higher age; 29.1%, 15.1%, 8.0% and 5.6% from the youngest to the oldest age group. The proportion living alone increased with higher age whilst it appeared quite similar between the ADL dependency groups.

Mortality rates amounted to 5.2% for the first 10 days after discharge, 8.3% for the first 30 days, and 14.3% for the first 90 days after discharge.

**Table 2:** Patient characteristics; overall average, per age group and degree of dependency. Standard deviation for continuous variables in parentheses

			All	< 70 years	70–79 years	80–89 years	*≥* 90 years	Slight/no dependency*	Moderate dependency*	Severe dependency*	Total dependency*
		Number of patients	8067	450	1954	3561	2102	388	2448	3103	2128
		Proportion of total (%)	100%	6%	24%	44%	26%	5%	30%	38%	26%
Demo-graphics		Sex, women (%)	62.7	53.3	56.0	62.7	70.7	53.4	64.3	64.7	59.5
		Age	83.5 (8.2)	66.1 (3.1)	75.1 (2.9)	84.7 (2.8)	93.1 (2.7)	80.3 (7.4)	82.7 (8.1)	84.0 (8.1)	84.4 (8.4)
Morbidity		Number of diagnoses	4.7 (1.8)	4.6 (1.8)	4.5 (1.9)	4.7 (1.8)	4.7 (1.8)	3.9 (1.7)	4.4 (1.7)	4.8 (1.8)	4.9 (1.9)
		Pharmaceuticals (continuous intake)	8.6 (4.2)	9.1 (5.1)	8.8 (4.4)	8.7 (4.1)	8.1 (3.9)	7.4 (3.9)	8.3 (4.0)	8.9 (4.2)	8.8 (4.3)
		Polypharmacy (%)	83.1	79.3	83.0	84.8	80.9	73.7	82.1	85.0	83.0
Risk screening		Barthel	52.1 (27.0)	55.5 (26.3)	55.7 (27.4)	53.0 (26.5)	46.6 (26.8)	97.2 (2.5)	76.7 (8.3)	44.0 (11.3)	10.9 (7.2)
		RMI	5.2 (3.7)	5.5 (3.9)	5.6 (3.9)	5.3 (3.6)	4.4 (3.4)	11.1 (2.5)	7.7 (2.6)	3.9 (2.5)	2.5 (3.3)
		Downton > 3 (%)	86.0	76.2	79.1	87.5	91.8	54.0	81.4	91.5	89.1
		Norton < 20 (%)	28.9	19.8	23.9	27.3	38.1	2.3	7.6	29.3	57.5
		MNA < 11 (%)	83.5	79.8	78.0	83.4	89.4	70.9	75.9	85.9	90.8
Socio-economics		Highest educational level (%)									
		* Primary*	22.0	7.6	17.3	23.8	26.7	17.7	21.8	22.2	22.8
		* Lower secondary*	12.8	23.4	14.2	11.3	11.6	13.5	13.1	12.5	12.6
		* Upper secondary*	36.2	36.2	35.6	35.0	38.8	33.1	36.1	36.8	36.1
		* Post-secondary*	19.4	22.0	21.7	20.1	15.4	22.2	20.5	19.0	18.2
		* Higher post-sec.*	9.6	10.8	11.2	9.8	7.5	13.5	8.5	9.6	10.3
		Annual disp. income, SEK	287 809	263 642	271 877	299 401	288 166	267 561	283 640	298 171	281 182
		Region of birth (%)									
		* Sweden*	82.6	81.3	81.8	81.2	86.2	84.0	81.9	82.8	82.9
		* Other*,* Nordic*	7.5	7.6	7.3	8.4	5.9	7.0	8.7	7.0	6.9
		* Other*,* Europe*	6.6	5.6	6.5	7.1	5.9	6.4	6.3	6.8	6.6
		* Outside Europe*	3.4	5.6	4.4	3.3	2.1	2.6	3.2	3.5	3.6
		Marital status (%)									
		*Married*	28.8	30.4	37.3	30.6	17.3	32.7	26.3	28.7	31.0
		*Widowed*	40.4	7.8	20.3	41.1	64.8	34.0	40.3	41.0	40.6
		*Never married*	10.3	29.1	15.1	8.0	5.6	10.3	10.8	10.1	10.0
		*Divorced*	20.6	32.7	27.3	20.3	12.4	22.9	22.7	20.2	18.4
		Living alone (%)	60.7	53.3	51.9	59.3	72.8	57.5	63.2	60.4	58.8

*Based on the Barthel index; cut-off values to categorize were < 20 (total dependency), 21–60 (severe dependency), 61–90 (moderate dependency), 91–100 (slight or no dependency)

### Service utilization

Table 3 presents health care utilization in terms of inpatient care days, outpatient visits, home-care service hours and days at RCF, for the study population overall as well as per group of age, physical function, and health condition.

Number of outpatient visits seemed to decrease with higher age, whilst number of home-care service hours and days at RCF (social care) were higher in the groups of higher age. Utilization of social care services was higher in groups with higher ADL dependency, as was inpatient care utilization. However, utilization of outpatient care was similar between groups of different ADL dependency.

The patterns of health care service utilization for the different diagnosis groups were varying. Individuals afflicted with cerebrovascular disease had the longest index inpatient care stay, whilst cancer and depression were conditions that showed significant additional use of inpatient care after the index admission. Individuals with dementia showed the highest levels of home-care services as well as the highest number of days at RCF, but also the lowest levels of total inpatient stay and outpatient visits. Utilization in terms of average outpatient visits per specialty care and primary care health care profession is presented in the supplementary tables, tables S2a and S2b.

The most common outpatient care contact was doctor visit at a care facility (hospital or other), for all categories and conditions included. The ratio of home visits to care facility visits, disregarding category of health personnel, was larger in primary care than in specialty care. The largest proportion of visits at home was delivered by assistant nurses in primary care.

**Table 3:** Service utilization for individuals in geriatric care; initial inpatient stay and six months of subsequent post-discharge health care and social care. Mean presented per age group, degree of dependency, and health condition. Confidence intervals (95%) in parentheses

	Initial inpatient stay (care days)	Additional inpatient stay (care days)	Outpatient specialty care (visits)	Outpatient primary care (visits)	Social services (hours)	Short-term care facility (days)	Long-term care facility (days)
< 70 years	9.1 (8.6;9.7)	5.3 (4.4;6.2)	8.9 (7.6;10.1)	30.4 (24.6;36.2)	209 (158;261)	2.0 (0.8;3.2)	9.0 (5.7;12.3)
70–79 years	8.9 (8.6;9.1)	4.1 (3.7;4.5)	6.5 (5.7;7.2)	29.5 (26.8;32.2)	315 (281;349)	3.1 (2.4;3.7)	14.7 (12.7;16.6)
80–89 years	9.1 (8.9;9.3)	3.6 (3.3;3.9)	4.3 (4.1;4.5)	29.2 (27.0;31.3)	440 (409;471)	3.3 (2.7;3.9)	25.1 (23.3;26.9)
*≥* 90 years	9.2 (9.0;9.4)	3.1 (2.8;3.4)	3.3 (3.1;3.5)	21.4 (19.0;23.7)	575 (529;621)	3.8 (3.1;4.6)	39.8 (37.0;42.6)
Slight/no dependency	5.4 (5.1;5.7)	3.1 (2.3;3.9)	5.6 (4.7;6.5)	20.5 (16.2;24.9)	104 (52;156)	0.3 (0.0;0.7)	4.5 (1.8;7.2)
Moderate dependency	7.5 (7.3;7.7)	3.6 (3.2;3.9)	4.7 (4.5;5.0)	28.0 (25.7;30.3)	226 (202;251)	2.0 (1.4;2.5)	10.7 (9.2;12.2)
Severe dependency	10.3 (10.1;10.5)	3.7 (3.4;4.0)	5.0 (4.5;5.4)	28.8 (26.6;30.9)	509 (475;543)	4.0 (3.4;4.6)	28.1 (26.1;30.1)
Total dependency	9.8 (9.5;10.1)	4.0 (3.6;4.3)	4.6 (4.2;4.9)	25.5 (22.6;28.4)	616 (566;665)	4.4 (3.6;5.3)	42.5 (40.0;45.3)
Cancer diagnosis	10.3 (9.6;11.0)	8.3 (6.5;10.0)	6.2 (5.1;7.2)	30.4 (23.8;37.1)	264 (177;352)	2.0 (0.4;3.7)	10.7 (6.5;15.0)
Chronic kidney disease	9.4 (7.5;11.3)	6.5 (2.7;10.3)	17.6 (7.0;28.3)	28.0 (11.3;44.6)	652 (290;1 013)	4.4 (0.0;8.8)	25.5 (8.5;42.4)
Dementia	8.4 (7.9;8.9)	2.2 (1.6;2.8)	3.2 (2.8;3.6)	31.1 (20.9;41.3)	861 (690;1 032)	6.9 (3.7;10.1)	61.8 (52.6;71.1)
Depression	8.4 (6.8;10.0)	9.8 (0.9;18.6)	6.1 (0.1;12.2)	28.4 (6.6;50.2)	679 (184;1 175)	2.9 (0.0;6.2)	22.8 (0.4;45.1)
Fragility fracture	10.6 (10.4;10.9)	2.7 (2.3;3.2)	5.2 (4.8;5.6)	23.6 (20.6;26.5)	470 (412;528)	3.1 (2.2;3.9)	28.7 (25.3;32.1)
Heart failure	8.8 (8.4;9.3)	5.2 (4.5;6.0)	4.1 (3.8;4.5)	33.4 (27.6;39.2)	367 (299;434)	2.2 (1.0;3.3)	20.2 (16.3;24.1)
Osteoporosis	10.4 (9.3;11.6)	3.8 (1.6;6.0)	4.6 (3.2;6.0)	19.4 (9.1;29.7)	488 (249;727)	5.0 (0.3;9.6)	27.1 (13.2;40.9)
Stroke/TIA	14.6 (13.7;15.5)	3.0 (2.2;3.9)	4.8 (3.9;5.6)	28.2 (22.5;34.0)	427 (323;530)	6.1 (3.5;8.7)	33.8 (26.8;40.8)
Average	9.1 (9.0;9.2)	3.7 (3.5;3.9)	4.8 (4.6;5.0)	27.3 (25.9;28.6)	432 (412;452)	3.3 (2.9;3.7)	25.5 (24.3;26.7)

### Estimation of costs

Table 4 shows health care and social care utilization translated to costs in Euro, based on unit costs presented in Table 1. The costs associated to service utilization for individuals in the oldest age group (≥ 90 years) were approximately 43% higher than those for individuals in the youngest age group (< 70 years). This cost difference corresponds to the absolute difference in home-care services utilization, whereas the incremental costs for RCF in the oldest group correspond to their lower utilization of inpatient and outpatient care. Dementia was the main diagnosis associated with the highest total costs for service utilization, driven by the highest degree of home-care services and RCF out of the health conditions included.

Comparing total costs between the different groups using ANOVA models showed that there were statistically significant differences in total costs between all groups of Barthel index; the higher dependency, the higher total costs. For age groups, there were statistically significant differences between all groups, except for between the two youngest. Regarding main diagnosis at the initial inpatient stay, dementia was significantly more costly than fragility fracture and heart failure, whilst stroke/TIA was significantly more costly than heart failure. Tables showing statistical significance at the 5% level for each comparison are included in the supplementary material, Tables S4a-c.

**Table 4:** Distribution of mean costs of care over age, degree of dependency, and main health condition, inflated to 2023 monetary value

(€)	Initial inpatient stay	Additional inpatient stay	Outpatient specialty care	Outpatient primary care	Social services	Short-term care facility	Long-term care facility	Total cost
< 70 years	17 286	10 068	4 900	5 744	11 995	574	2 582	53 149
70–79 years	16 906	7 788	3 654	5 537	18 047	889	4 217	57 038
80–89 years	17 286	6 838	2 459	5 194	25 181	947	7 200	65 106
*≥* 90 years	17 476	5 889	1 927	3 776	32 917	1 090	11 417	74 491
*Proportion*	*26*,*8%*	*11*,*1%*	*4*,*4%*	*7*,*8%*	*37*,*5%*	*1*,*5%*	*10*,*9%*	*-*
Slight/no dependency	10 258	5 889	3 046	3 887	5 966	86	1 291	30 422
Moderate dependency	14 247	6 838	2 717	5 078	12 963	574	3 069	45 486
Severe dependency	19 565	7 028	2 803	5 281	29 155	1 147	8 061	73 040
Total dependency	18 616	7 598	2 630	4 472	35 247	1 262	12 191	82 016
*Proportion*	*27*,*4%*	*11*,*6%*	*4*,*6%*	*8*,*2%*	*36*,*3%*	*1*,*4%*	*10*,*4%*	*-*
Cancer diagnosis	19 565	15 766	3 653	5 590	15 144	574	3 069	63 362
Chronic kidney disease	17 856	12 347	9 002	5 509	37 326	1 233	7 315	90 587
Dementia	15 956	4 179	1 911	4 419	49 298	1 979	17 728	95 470
Depression	15 956	18 616	3 215	5 096	38 900	832	6 540	89 156
Fragility fracture	20 135	5 129	2 995	4 655	26 916	889	8 233	68 952
Heart failure	16 716	9 878	2 378	5 850	20 996	631	5 794	62 243
Osteoporosis	19 755	7 218	2 692	3 928	27 936	1 434	7 774	70 737
Stroke/TIA	27 733	5 699	2 711	6 012	24 426	1 750	9 696	78 027
Average	17 237	7 030	2 743	4 938	24 735	947	7 315	64 944
*Proportion*	*26.5%*	*10.8%*	*4.2%*	*7.6%*	*38.1%*	*1.5%*	*11.3%*	*-*

Arranging the categories in order based on total costs incurred (Table S5), shows that higher age seemed positively correlated with higher total cost, and higher degree of dependency seemed positively correlated with higher total cost. Dementia was estimated as the most expensive health condition of the ones included, followed by chronic kidney disease and depression. For all conditions with the highest total cost, their largest cost bucket was that of social services.

## Discussion

### Service utilization

In the present study, there were substantial differences between patient sub-groups in terms of types of service utilized, i.e. health care or social care, which are financed by different authorities. Furthermore, there were meaningful differences between the “intensity” of services leveraged, i.e. inpatient (higher intensity) versus outpatient care (lower intensity) or staying at an RCF (higher) versus home-care services (lower), as well as in the relative levels between these. Geriatric care is a specialty covering various health conditions and individuals with different degrees of dependency, and medical and social demands likely vary between the different sub-groups. Some sub-groups tended to utilize health care to a higher extent relative to their utilization of social care, e.g. cancer patients, and the group younger than 70 years, whilst the opposite was the case for several groups including e.g. dementia, and the group with highest degree of dependency (Barthel index < 20).

Previous studies on older adults and utilization of health care services have generally assessed care burden relating to particular conditions, where older adults are often one or few of several age categories included in the study [32, 33]. There are also dedicated studies on older adults for particular conditions [7–9]. However, we have not found studies assessing the care burden for individuals with history of geriatric inpatient care or for older adults in general, nor studies of particular conditions assessing the burden on health care and social care as well.

### Estimation of costs

The regional costs for inpatient care outweighed outpatient costs (on average 24.3 thousand EUR versus 7.7) for this study population where everyone had had at least one inpatient stay. For the municipalities, the relationship was in a way the opposite; home-care social services were more costly, compared to RCF (on average 24.7 thousand EUR versus 8.3). The average cost split between region and municipality was even; 49% versus 51% over the six months studied. Costs for outpatient specialty care were predominantly driven by doctor visits, whilst the most costly category in primary care was home visits by assistant nurses.

It is challenging to compare studies of costing between contexts. One of the most recent studies on the subject of costs for care of the geriatric population was performed in the Netherlands for a population of 401 older adults acutely hospitalized during 2015–2017 [34]. The 90-day costs after discharge were estimated to Euro 4 035 which represents just a fraction of the costs estimated in the present study. Likely the differences in results can partly be explained by the sampling as well as cost categories included; the referenced study included older adults with an emergency department visit, whilst the present study included only individuals with an inpatient stay at a geriatric department. Furthermore, the referenced study included costs for RCF stay but not for any additional social care.

Health care and social care are almost entirely covered by taxes. There is a ceiling for out-of-pocket (OOP) outpatient health care costs at 1,400 Swedish kronor or 126 Euro per year (free of charge for individuals above 85 years of age), and the patient OOP cost for inpatient care amounts to 130 Swedish kronor or 12 Euro per night [35]. The ceiling for social care costs is set to 2,359 Swedish kronor (212 Euro) for home-care social services, and 2,423 Swedish kronor (218 Euro) for stay at RCF, per month. All in all, OOP costs can be considered being of negligible magnitude in a total costs’ perspective.

The finding that dementia stands out as the most costly of the groups of main diagnosis studied here is not new; costs of dementia have been extensively assessed in previous research. Cost estimates have been found to differ considerably depending on the differences in health care systems within different countries studied, one reason pointed out as variability of included categories of costs [36] which is likely not limited to studies of dementia. It has also been pointed out that level of function may better explain differences in care costs than for example the individual’s diagnosis [37]. In addition to costs associated to formal dementia care mentioned here, informal care has been estimated to make up half of the global costs of dementia [38].

### Methodological considerations

There are challenges in estimating costs for specific health conditions or diagnoses, as for example dementia mentioned above. One reason is that the group of patients studied is often afflicted with several chronic conditions simultaneously, and it is a delicate task to disentangle separate work streams of specialty care to estimate costs for only one particular condition. Such estimations could also end up being misleading, as care delivery for different health conditions are interdependent. In the present study the selected health conditions, found amongst the most common diagnoses registered, were defined based on main diagnosis (supplementary table S1) during the index admission. Main diagnosis was leveraged instead of any secondary diagnosis, as this approach was expected to better reflect service utilization related to these selected conditions more than if each health condition registered as a secondary diagnosis would be the basis of cost allocation. Still, the cost estimations do not reflect care related only to one particular health condition but additional care for additional health conditions as well. Leveraging an approach that consider function, i.e. ADL dependency, to estimate costs instead, is likely in general a more precise method.

An important feature regarding the patient group studied here, is the dependency on more than one formal financer of care; the regional health care system and the municipal social care system. Some sub-groups within the geriatric population are in relatively higher need of the former, and some of the latter. The balance of costs between these two financers differs depending on the individual’s health condition and thereby the need for different kinds of care. In a well-functioning system this balance would not impact differences in quality of care, i.e. if the care transitions between the financers ran smoothly. However, a significant risk lies within the care transition per se if the transitions are done with e.g. loss of information as a result. In such a scenario, the total costs for a patient’s care journey are likely higher. Thus, further analysis of total costs and the feasibility of their levels need to be performed simultaneously with an assessment of the transitions between the two financers.

An additional challenge in studying a group of older adults is how to approach the non-negligible mortality during the follow-up (14.3% during the first 90 days). In the present study, all individuals alive at discharge were included in order to report a mean cost for them. Alternative scenarios would be to exclude later-on deceased individuals, but this would likely skew the results inappropriately.

Regarding potential generalizability of this study, the cohort studied here has been compared to the region’s general population of older adults with a history of geriatric inpatient care, and was found to be a representative sample regarding utilization of hospital care, however less representative regarding primary care utilization [18]. This should be considered in any further analyses relating to utilization of primary care for this group.

### Strengths and limitations

This is the first estimate of service utilization and costs including health care as well as social care for the population of individuals with assessed need of geriatric inpatient care in Sweden. This is a registry-based study with the aim to estimate service utilization and costs for a Swedish study population receiving geriatric inpatient care, and it contributes with a detailed perspective on utilization in terms of leveraged service units as well as direct costs, relating to overall care for this patient group.

With a pseudonymized variant of the personal identity number, individual-level linking of data was made possible, and cost estimations based on units of service and their actual costs reported from care givers were performed. One additional major strength with the present study is that population registries leveraged generally do have complete coverage. A limitation related to the registries is that it may be precarious to use databases developed for administrative and follow-up purposes to perform cost analysis such as in the present study. Furthermore, there are various registers available with various quality of data, but the general quality and validity of the population-based registers are high [39] and continues to be our best available tool for retrospective cohort studies [40]. An additional limitation in this context is that the data leveraged are a few years old, which means that there is a risk that new ways of working, organizational changes and updated routines and legislation may have had an impact that is not detectable within the results presented in this study. One example of such changes is updated legislation on collaboration at discharge which has been subject to updated routines in the context of this study population, aiming to strengthen the continuity after discharge from inpatient care [41].

Consideration was not taken to costs outside of health care and social care, such as informal care and work absence. It may be worth mentioning– in an international perspective– that all individuals in the Swedish context have the same right to social care such as home-care services, no matter if there is a family that can help out, or not. With that said, the indirect economic burden of informal care is likely of substantial size. Indirect costs due to work absence for the individual is expected to be low due to the high age of the study population, yet higher for individuals delivering the informal care; depending on the amount of informal care delivered for the study population, the total costs of care are likely significantly higher than presented here. Costs for pharmaceuticals after discharge from inpatient care also lack, and hence the overall direct costs related to care are somewhat higher than the estimates presented here. The costs for staying at RCF are based on CPU estimates relating to e.g. occupancy rates and personnel density, but it is however not stated whether health personnel (e.g. physiotherapists and nurses) employed by the municipality are included in these estimates or not. If not, health care costs for individuals living at RCF may be underestimated.

### Further research

The inclusion of data on Health-Related Quality of Life would further strengthen the potential use of the estimations in addition to measures of function and ADL dependency used in the present study. This could be done via patient-reported outcome measures developed and validated for this group specifically [42]. Furthermore, the informal care delivered to the individuals receiving geriatric inpatient care, and its costs, need to be further investigated in order to complete the picture. It has not been possible to compare the differences in service utilization and costs between municipalities. This would however be of significant interest to understand better, as the social care offered after discharge is dependent on an assessment made by the municipality representative. Such an assessment may long-term imply differences in need in terms of other services such as those from regional health care.

## Conclusions

Individuals admitted to geriatric inpatient care spent on average approximately 13 inpatient care days during a six-month period, had five visits in specialty care, 27 visits in primary care, 430 hours of home-care services, and 29 days of staying at RCF. This equalled approximately 65 thousand Euros, on average, but ranged on group-level from 30 thousand to 82 thousand depending on ADL dependency, and home-based health care is also included in these estimates. The estimated costs incurred were made up to 49% financed by the region, and 51% financed by the municipality.

The group of individuals in need of geriatric care is heterogeneous, and service utilization varies significantly between groups of age, degree of ADL dependency, and underlying health condition. Also, the cost burden per payer (region or municipality) varies significantly between these groups. Hence, different groups show differences in articulated demands and received care towards the region and the municipality.

These results should work as estimates for further analysis and not as levels to steer towards, as utilization does not always reflect actual needs, which may or may not be in fact higher.

## Electronic supplementary material

Below is the link to the electronic supplementary material.

Supplementary Material 1: **Table S1**. ICD-10 codes used for main diagnosis categorization. **Table S2a-b**. Outpatient service utilization, per health care personnel category. **Table S3a-b**. Costs of outpatient visits, per health care personnel category. **Table S4a-c**. Overview of post-hoc ANOVA model tests between groups. **Table S5**. Overview of costs of care per group of categorization. **Table S6**. Prevalence of health conditions per group of age and physical function. **Fig. S1**. Flow chart of study population and exclusions made.

## Data Availability

Data is available upon request, and requests for access to the data can be sent to the Research Data Office (rdo@ki.se) at Karolinska Institutet. RDO is a virtual organization with staff from the Archive, the Compliance and Data Office, the IT department (ITA) and the University Library (KIB). The request will be handled according to relevant legislation. This will require a data processing agreement or similar with the recipient of the data. The reason for this procedure is legal (in accordance with the General Data Protection Regulation) and relates to the high sensitivity of the data and the potential harm to the individual should the data be publicly known.
