# Supplementary material for: Exploration of health care utilization, social care utilization and costs for individuals discharged from inpatient geriatric care in Sweden - a registry data study
Source: Health Econ Rev. 2025 Mar 13;15:18. doi: 10.1186/s13561-025-00610-1 (PMC11905552; doi:10.1186/s13561-025-00610-1)

Table S1. ICD-10-SE codes used for main diagnosis categorization.

| **Health condition** | **ICD-10-SE codes** |
| --- | --- |
| Cancer diagnosis | C* |
| Chronic kidney disease | I12.0, I13.1, N03.2-N03.7, N05.2-N05.7, N18, N19, N25.0, Z49.0,-Z49.2, Z94.0, Z99.2 |
| Dementia | F00-F03, F05.1, G30, G31.1 |
| Depression | F20.4, F31.3, F31.4, F31.5, F32, F33, F34.1, F41.2, F43.2 |
| Fragility fracture | M48.5, M80.0, M80.8, M84.3-M84.7, S02, S12, S22, S32, S42, S52, S62, S72, S82, S92 |
| Heart failure | I09.9, I11.0, I13.0, I13.2, I25.5, I42.0, I42.5, I42.6, I42.7, I42.8, I42.9, I43, I50, P29.0 |
| Osteoporosis | M80, M81 |
| Stroke/TIA | G45.0-G45.3, G45.8, G45.9, H34.1, I60, I61, I63, I64 |

Table S2a. Outpatient health care service utilization (specialty care) during the first six months after discharge from geriatric hospital care; average number of single visits per health care personnel category. Advanced home care (ASIH) included within primary care, table S2b.

|  | **Specialty care doctor (visits)** | **Specialty care doctor (home visits)** | **Specialty care nurse (visits)** | **Specialty care nurse (home visits)** | **Specialty care assistant nurse (visits)** | **Specialty care assistant nurse (home visits)** | **Specialty care OT (visits)** | **Specialty care OT (home visits)** | **Specialty care PT (visits)** | **Specialty care PT (home visits)** | **Specialty care other (visits)** | **Specialty care other (home visits)** | **Team (visits and home visits)** |
| --- | --- | --- | --- | --- | --- | --- | --- | --- | --- | --- | --- | --- | --- |
| <70 years | 4.9 | 0.3 | 1.8 | 0.2 | 0.1 | 0.0 | 0.1 | 0.1 | 0.5 | 0.2 | 0.2 | 0.1 | 0.4 |
| 70-79 years | 3.8 | 0.5 | 1.4 | 0.1 | 0.0 | 0.0 | 0.0 | 0.0 | 0.3 | 0.0 | 0.1 | 0.0 | 0.2 |
| 80-89 years | 2.7 | 0.8 | 0.5 | 0.0 | 0.0 | 0.0 | 0.0 | 0.0 | 0.1 | 0.0 | 0.1 | 0.0 | 0.1 |
| >90 years | 1.7 | 1.2 | 0.2 | 0.0 | 0.0 | 0.0 | 0.0 | 0.0 | 0.1 | 0.0 | 0.0 | 0.0 | 0.0 |
| Slight/no dependency | 3.6 | 0.1 | 1.1 | 0.0 | 0.0 | 0.0 | 0.0 | 0.1 | 0.2 | 0.1 | 0.1 | 0.0 | 0.1 |
| Moderate dependency | 3.2 | 0.4 | 0.6 | 0.1 | 0.0 | 0.0 | 0.0 | 0.0 | 0.1 | 0.0 | 0.1 | 0.0 | 0.1 |
| Severe dependency | 2.7 | 0.9 | 0.8 | 0.0 | 0.0 | 0.0 | 0.0 | 0.0 | 0.2 | 0.0 | 0.1 | 0.0 | 0.1 |
| Total dependency | 2.5 | 1.2 | 0.5 | 0.0 | 0.0 | 0.0 | 0.0 | 0.0 | 0.1 | 0.0 | 0.1 | 0.0 | 0.1 |
| Cancer diagnosis | 3.2 | 0.5 | 1.2 | 0.0 | 0.0 | 0.0 | 0.1 | 0.0 | 0.4 | 0.0 | 0.2 | 0.0 | 0.6 |
| Chronic kidney disease | 6.8 | 1.4 | 8.4 | 0.0 | 0.1 | 0.0 | 0.0 | 0.0 | 0.1 | 0.0 | 0.9 | 0.0 | 0.1 |
| Dementia | 1.5 | 1.4 | 0.1 | 0.0 | 0.0 | 0.0 | 0.0 | 0.0 | 0.0 | 0.0 | 0.0 | 0.0 | 0.1 |
| Depression | 2.2 | 0.6 | 3.0 | 0.0 | 0.0 | 0.0 | 0.0 | 0.0 | 0.1 | 0.0 | 0.1 | 0.0 | 0.2 |
| Fragility fracture | 3.4 | 0.8 | 0.5 | 0.0 | 0.0 | 0.0 | 0.1 | 0.0 | 0.3 | 0.0 | 0.1 | 0.0 | 0.1 |
| Heart failure | 2.6 | 0.7 | 0.6 | 0.0 | 0.0 | 0.0 | 0.0 | 0.0 | 0.1 | 0.0 | 0.0 | 0.0 | 0.1 |
| Osteoporosis | 2.7 | 1.0 | 0.2 | 0.5 | 0.0 | 0.1 | 0.0 | 0.0 | 0.0 | 0.0 | 0.0 | 0.0 | 0.0 |
| Stroke/TIA | 2.6 | 1.2 | 0.7 | 0.0 | 0.0 | 0.0 | 0.0 | 0.0 | 0.1 | 0.0 | 0.1 | 0.0 | 0.0 |
| Average | 2.8 | 0.8 | 0.7 | 0.0 | 0.0 | 0.0 | 0.0 | 0.0 | 0.2 | 0.0 | 0.1 | 0.0 | 0.1 |

Table S2b. Outpatient health care service utilization (primary care) during the first six months after discharge from geriatric hospital care; average number of single visits per health care personnel category. Includes advanced home care (ASIH).

|  | **Primary care doctor (visits)** | **Primary care doctor (home visits)** | **Primary care nurse (visits)** | **Primary care nurse (home visits)** | **Primary care assistant nurse (visits)** | **Primary care assistant nurse (home visits)** | **Primary care OT (visits)** | **Primary care OT (home visits)** | **Primary care PT (visits)** | **Primary care PT (home visits)** | **Primary care other (visits)** | **Primary care other (home visits)** | **Team (visits and home visits)** |
| --- | --- | --- | --- | --- | --- | --- | --- | --- | --- | --- | --- | --- | --- |
| <70 years | 3.4 | 0.6 | 0.4 | 6.6 | 0.4 | 12.5 | 0.1 | 1.2 | 1.4 | 1.6 | 0.2 | 0.5 | 1.4 |
| 70-79 years | 3.9 | 0.5 | 0.3 | 6.0 | 0.1 | 12.4 | 0.2 | 1.1 | 1.5 | 1.7 | 0.1 | 0.5 | 1.3 |
| 80-89 years | 3.8 | 0.4 | 0.2 | 4.6 | 0.1 | 15.6 | 0.1 | 0.9 | 0.5 | 1.3 | 0.1 | 0.5 | 1.1 |
| >90 years | 2.5 | 0.4 | 0.1 | 3.7 | 0.0 | 11.8 | 0.0 | 0.7 | 0.2 | 0.8 | 0.0 | 0.3 | 0.8 |
| Slight/no dependency | 5.2 | 0.3 | 0.5 | 3.1 | 0.1 | 7.9 | 0.1 | 0.5 | 1.1 | 0.6 | 0.2 | 0.3 | 0.5 |
| Moderate dependency | 4.6 | 0.4 | 0.3 | 4.2 | 0.1 | 13.8 | 0.1 | 0.8 | 1.0 | 1.2 | 0.1 | 0.4 | 1.0 |
| Severe dependency | 3.2 | 0.5 | 0.2 | 5.5 | 0.1 | 14.0 | 0.1 | 1.1 | 0.7 | 1.7 | 0.1 | 0.4 | 1.3 |
| Total dependency | 2.1 | 0.4 | 0.1 | 4.8 | 0.1 | 14.1 | 0.1 | 0.8 | 0.4 | 1.0 | 0.1 | 0.5 | 1.0 |
| Cancer diagnosis | 1.7 | 1.1 | 0.3 | 14.2 | 0.1 | 9.4 | 0.0 | 1.0 | 0.2 | 1.2 | 0.0 | 0.5 | 0.6 |
| Chronic kidney disease | 2.3 | 0.5 | 0.1 | 12.7 | 0.0 | 8.7 | 0.1 | 0.8 | 0.0 | 0.6 | 0.1 | 0.2 | 1.8 |
| Dementia | 2.0 | 0.2 | 0.1 | 1.8 | 0.0 | 25.3 | 0.0 | 0.5 | 0.1 | 0.5 | 0.0 | 0.2 | 0.5 |
| Depression | 4.3 | 0.2 | 0.2 | 3.9 | 0.0 | 16.1 | 0.0 | 0.6 | 0.4 | 0.4 | 0.4 | 0.5 | 1.4 |
| Fragility fracture | 3.4 | 0.3 | 0.2 | 3.4 | 0.1 | 9.5 | 0.1 | 1.0 | 1.3 | 2.5 | 0.1 | 0.3 | 1.4 |
| Heart failure | 4.6 | 0.7 | 0.2 | 8.0 | 0.2 | 16.7 | 0.0 | 0.8 | 0.2 | 0.7 | 0.0 | 0.3 | 0.9 |
| Osteoporosis | 4.0 | 0.3 | 0.1 | 3.0 | 0.0 | 7.6 | 0.0 | 1.0 | 0.3 | 1.7 | 0.1 | 0.3 | 0.9 |
| Stroke/TIA | 2.5 | 0.4 | 0.1 | 2.8 | 0.0 | 10.9 | 0.3 | 2.5 | 1.1 | 3.2 | 0.3 | 2.4 | 1.7 |
| Average | 3.5 | 0.4 | 0.2 | 4.8 | 0.1 | 13.7 | 0.1 | 0.9 | 0.7 | 1.3 | 0.1 | 0.4 | 1.1 |

Table S3a. Costs of outpatient visits, per health care personnel category.

| **(€)** | **Specialty care doctor** | **Specialty care doctor (home)** | **Specialty care nurse** | **Specialty care nurse (home)** | **Specialty care assistant nurse** | **Specialty care assistant nurse (home)** | **Specialty care OT** | **Specialty care OT (home)** | **Specialty care PT** | **Specialty care PT (home)** | **Specialty care other** | **Specialty care other (home)** | **Team** |
| --- | --- | --- | --- | --- | --- | --- | --- | --- | --- | --- | --- | --- | --- |
| <70 years | 2 581 | 168 | 660 | 87 | 14 | 0 | 26 | 16 | 152 | 30 | 69 | 24 | 352 |
| 70-79 years | 1 996 | 230 | 491 | 30 | 10 | 0 | 10 | 3 | 90 | 2 | 49 | 2 | 204 |
| 80-89 years | 1 397 | 383 | 182 | 9 | 6 | 0 | 6 | 3 | 34 | 0 | 21 | 0 | 56 |
| >90 years | 905 | 569 | 63 | 17 | 2 | 2 | 3 | 3 | 19 | 1 | 16 | 2 | 43 |
| Slight/no dependency | 1 869 | 58 | 404 | 1 | 5 | - | 8 | 15 | 56 | 21 | 53 | 14 | 93 |
| Moderate dependency | 1 654 | 186 | 232 | 29 | 6 | 2 | 8 | 3 | 42 | 0 | 33 | 0 | 120 |
| Severe dependency | 1 425 | 446 | 289 | 13 | 8 | 0 | 8 | 4 | 67 | 2 | 23 | 3 | 102 |
| Total dependency | 1 287 | 574 | 195 | 24 | 4 | 0 | 5 | 2 | 35 | 1 | 30 | 3 | 81 |
| Cancer diagnosis | 1 671 | 223 | 450 | 14 | 9 | - | 12 | - | 106 | - | 65 | - | 565 |
| Chronic kidney disease | 3 572 | 670 | 3 040 | - | 13 | - | - | - | 22 | - | 312 | - | 46 |
| Dementia | 798 | 712 | 49 | 2 | - | - | 1 | 2 | - | - | 10 | - | 56 |
| Depression | 1 133 | 306 | 1 088 | - | - | - | - | - | 20 | - | 37 | - | 157 |
| Fragility fracture | 1 753 | 399 | 169 | 15 | 12 | 0 | 15 | 3 | 75 | 1 | 19 | - | 93 |
| Heart failure | 1 355 | 370 | 207 | 2 | 1 | 0 | 2 | 2 | 18 | 0 | 17 | 1 | 53 |
| Osteoporosis | 1 413 | 500 | 65 | 301 | - | 6 | 7 | - | 4 | - | - | - | - |
| Stroke/TIA | 1 373 | 584 | 247 | 4 | 5 | 0 | 2 | - | 19 | - | 49 | - | 28 |
| Average | 1 480 | 382 | 253 | 20 | 6 | 1 | 7 | 4 | 50 | 2 | 29 | 2 | 102 |

Table S3b. Costs of outpatient visits, per health care personnel category.

|  | **Primary care doctor** | **Primary care doctor (home)** | **Primary care nurse** | **Primary care nurse (home)** | **Primary care assistant nurse** | **Primary care assistant nurse (home)** | **Primary care OT** | **Primary care OT (home)** | **Primary care PT** | **Primary care PT (home)** | **Primary care other** | **Primary care other** | **Team** |
| --- | --- | --- | --- | --- | --- | --- | --- | --- | --- | --- | --- | --- | --- |
| <70 years | 750 | 169 | 44 | 1 009 | 41 | 1 221 | 10 | 313 | 187 | 308 | 39 | 151 | 655 |
| 70-79 years | 850 | 132 | 32 | 912 | 10 | 1 216 | 18 | 273 | 197 | 321 | 25 | 149 | 585 |
| 80-89 years | 832 | 123 | 22 | 701 | 7 | 1 531 | 6 | 229 | 71 | 250 | 11 | 131 | 515 |
| >90 years | 549 | 118 | 11 | 566 | 5 | 1 155 | 3 | 167 | 20 | 153 | 2 | 85 | 384 |
| Slight/no dependency | 1 146 | 72 | 50 | 480 | 17 | 776 | 14 | 132 | 145 | 116 | 28 | 89 | 248 |
| Moderate dependency | 1 009 | 125 | 31 | 639 | 11 | 1 355 | 10 | 206 | 128 | 222 | 19 | 124 | 449 |
| Severe dependency | 712 | 137 | 21 | 839 | 8 | 1 367 | 9 | 268 | 94 | 317 | 10 | 120 | 599 |
| Total dependency | 465 | 123 | 12 | 737 | 7 | 1 384 | 6 | 213 | 47 | 190 | 10 | 137 | 482 |
| Cancer diagnosis | 382 | 310 | 29 | 2 171 | 8 | 920 | 0 | 260 | 31 | 233 | 5 | 147 | 272 |
| Chronic kidney disease | 507 | 154 | 13 | 1 945 | - | 856 | 6 | 206 | 3 | 115 | 10 | 59 | 824 |
| Dementia | 444 | 46 | 8 | 271 | 0 | 2 475 | 0 | 124 | 11 | 84 | 3 | 67 | 234 |
| Depression | 939 | 59 | 21 | 601 | - | 1 574 | - | 156 | 60 | 71 | 71 | 150 | 646 |
| Fragility fracture | 738 | 95 | 19 | 519 | 9 | 930 | 12 | 238 | 179 | 476 | 9 | 89 | 655 |
| Heart failure | 1 016 | 208 | 22 | 1 223 | 25 | 1 637 | 2 | 193 | 32 | 138 | 4 | 77 | 412 |
| Osteoporosis | 881 | 95 | 8 | 455 | 2 | 743 | 5 | 258 | 39 | 323 | 10 | 97 | 435 |
| Stroke/TIA | 548 | 114 | 14 | 422 | 5 | 1 066 | 34 | 637 | 148 | 591 | 55 | 703 | 791 |
| Average | 758 | 127 | 23 | 734 | 9 | 1 340 | 9 | 228 | 94 | 245 | 14 | 124 | 506 |

Table S4a. Post-hoc ANOVA model tests between total costs per age group; asterisks mark statistical significance of p<0,05.

|  | <70 years | 70-79 years | 80-89 years | >90 years |
| --- | --- | --- | --- | --- |
| <70 years |  |  | *** | *** |
| 70-79 years |  |  | *** | *** |
| 80-89 years | *** | *** |  | *** |
| >90 years | *** | *** | *** |  |

Table S4b. Post-hoc ANOVA model tests between total costs per Barthel group; asterisks mark statistical significance of p<0,05.

|  | Slight/no dependency | Moderate dependency | Severe dependency | Total dependency |
| --- | --- | --- | --- | --- |
| Slight/no dependency |  | *** | *** | *** |
| Moderate dependency | *** |  | *** | *** |
| Severe dependency | *** | *** |  | *** |
| Total dependency | *** | *** | *** |  |

Table S4c. Post-hoc ANOVA model tests between total costs per diagnosis group; asterisks mark statistical significance of p<0,05.

|  | Cancer diagnosis | Chronic kidney disease | Dementia | Depression | Fragility fracture | Heart failure | Osteoporosis | Stroke/TIA |
| --- | --- | --- | --- | --- | --- | --- | --- | --- |
| Cancer diagnosis |  |  | *** |  |  |  |  |  |
| Chronic kidney disease |  |  |  |  |  |  |  |  |
| Dementia | *** |  |  |  | *** | *** |  |  |
| Depression |  |  |  |  |  |  |  |  |
| Fragility fracture |  |  | *** |  |  |  |  |  |
| Heart failure |  |  | *** |  |  |  |  | *** |
| Osteoporosis |  |  |  |  |  |  |  |  |
| Stroke/TIA |  |  |  |  |  | *** |  |  |

Table S5. Costs of care for individuals in geriatric care during the first six months after discharge from hospital care; selected conditions and overall average. Inflated to 2023 monetary value. For each row (patient category) the nuances reflect lower (light grey) and higher (dark grey) costs respectively, attributable to the different service categories for that patient category. The groups are arranged from higher to lower based on the far-right column of total costs.

| **(€)** | Inpatient care | Outpatient care | Social services | Residential care | Total |
| --- | --- | --- | --- | --- | --- |
| Dementia | 20 135 | 6 330 | 49 298 | 19 707 | 95 470 |
| Chronic kidney disease | 30 203 | 14 511 | 37 326 | 8 548 | 90 587 |
| Depression | 34 572 | 8 312 | 38 900 | 7 372 | 89 156 |
| Total dependency | 26 214 | 7 101 | 35 247 | 13 454 | 82 016 |
| Stroke/TIA | 33 432 | 8 724 | 24 426 | 11 446 | 78 027 |
| Aged >90 years | 23 364 | 5 703 | 32 917 | 12 507 | 74 491 |
| Severe dependency | 26 594 | 8 084 | 29 155 | 9 208 | 73 040 |
| Osteoporosis | 26 974 | 6 620 | 27 936 | 9 208 | 70 737 |
| Fragility fracture | 25 264 | 7 649 | 26 916 | 9 122 | 68 952 |
| Aged 80-89 years | 24 124 | 7 654 | 25 181 | 8 147 | 65 106 |
| Cancer diagnosis | 35 332 | 9 243 | 15 144 | 3 643 | 63 362 |
| Heart failure | 26 594 | 8 228 | 20 996 | 6 426 | 62 243 |
| Aged 70-79 years | 24 694 | 9 191 | 18 047 | 5 106 | 57 038 |
| Aged <70 years | 27 353 | 10 645 | 11 995 | 3 155 | 53 149 |
| Moderate dependency | 21 085 | 7 795 | 12 963 | 3 643 | 45 486 |
| Slight/no dependency | 16 146 | 6 933 | 5 966 | 1 377 | 30 422 |
| Average | 24 266 | 7 681 | 24 735 | 8 261 | 64 944 |

Table S6. Prevalence of health conditions per group of age and physical function. Based on main diagnosis during index admission.

| **All** | **<70 years** | **70-79 years** | **80-89 years** | **>90 years** | **Slight/no dependency*** | **Moderate dependency*** | **Severe dependency*** | **Total dependency*** |
| --- | --- | --- | --- | --- | --- | --- | --- | --- |
| Cancer diagnosis | 5,3% | 3,7% | 3,2% | 2,0% | 3,1% | 3,5% | 3,3% | 2,4% |
| Chronic kidney disease | 0,7% | 0,4% | 0,4% | 0,7% | 0,0% | 0,4% | 0,5% | 0,6% |
| Dementia | 0,9% | 2,3% | 3,8% | 2,9% | 2,1% | 2,2% | 3,2% | 3,9% |
| Depression | 0,2% | 0,3% | 0,5% | 0,1% | 0,8% | 0,3% | 0,4% | 0,4% |
| Fragility fracture | 15,6% | 17,5% | 13,8% | 11,0% | 2,6% | 9,3% | 20,5% | 12,4% |
| Heart failure | 3,3% | 4,6% | 7,0% | 11,5% | 8,8% | 9,1% | 6,4% | 6,5% |
| Osteoporosis | 1,1% | 0,6% | 1,1% | 0,9% | 0,0% | 1,1% | 1,1% | 0,6% |
| Stroke/TIA | 3,3% | 5,0% | 3,9% | 2,5% | 2,1% | 3,5% | 2,9% | 5,7% |

*Based on the Barthel index; cut-off values to categorize were <20 (total dependency), 21-60 (severe dependency), 61-90 (moderate dependency), 91-100 (slight or no dependency).

Figure S1. Flow chart of study population and exclusions made.


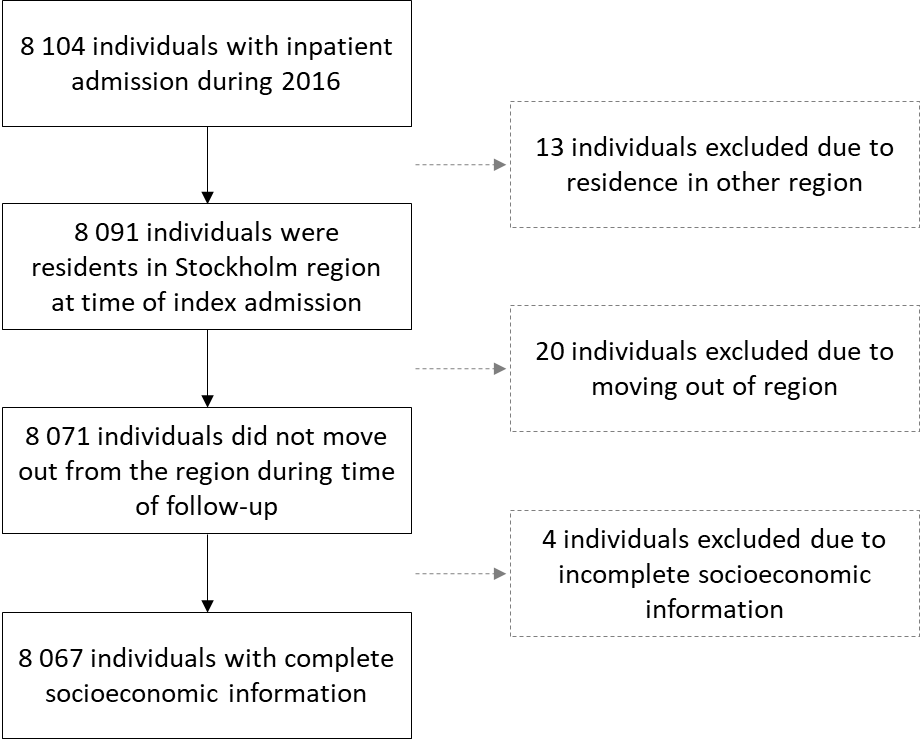

Supplement: Supplementary file 1 — Supplementary Material 1: Table S1. ICD-10 codes used for main diagnosis categorization. Table S2a-b. Outpatient service utilization, per health care personnel category. Table S3a-b. Costs of outpatient visits, per health care personnel category. Table S4a-c. Overview of post-hoc ANOVA model tests between groups. Table S5. Overview of costs of care per group of categorization. Table S6. Prevalence of health conditions per group of age and physical function. Fig. S1. Flow chart of study population and exclusions made. [file 13561_2025_610_MOESM1_ESM.docx]
